# Identification of Kv4.2 protein complex and modifications by tandem affinity purification-mass spectrometry in primary neurons

**DOI:** 10.3389/fncel.2022.1070305

**Published:** 2022-12-09

**Authors:** Jia-Hua Hu, Ying Liu, Dax A. Hoffman

**Affiliations:** Section on Molecular Neurophysiology and Biophysics, Eunice Kennedy Shriver National Institute of Child Health and Human Development, Bethesda, MD, United States

**Keywords:** tandem affinity purification (TAP), Kv4.2 (KCND2), protein complex, neurons, phosphorylation

## Abstract

Proteins usually form complexes to fulfill variable physiological functions. In neurons, communication relies on synapses where receptors, channels, and anchoring proteins form complexes to precisely control signal transduction, synaptic integration, and action potential firing. Although there are many published protocols to isolate protein complexes in cell lines, isolation in neurons has not been well established. Here we introduce a method that combines lentiviral protein expression with tandem affinity purification followed by mass-spectrometry (TAP-MS) to identify protein complexes in neurons. This protocol can also be used to identify post-translational modifications (PTMs) of synaptic proteins. We used the A-type voltage-gated K^+^ channel subunit Kv4.2 as the target protein. Kv4.2 is highly expressed in the hippocampus where it contributes to learning and memory through its regulation of neuronal excitability and synaptic plasticity. We tagged Kv4.2 with the calmodulin-binding-peptide (CBP) and streptavidin-binding-peptide (SBP) at its C-terminus and expressed it in neurons *via* lentivirus. Kv4.2 was purified by two-step TAP and samples were analyzed by MS. MS identified two prominently known Kv4.2 interacting proteins [dipeptidyl peptidase like (DPPs) and Kv channel-interacting proteins (KChIPs)] in addition to novel synaptic proteins including glutamate receptors, a calcium channel, and anchoring proteins. Co-immunoprecipitation and colocalization experiments validated the association of Kv4.2 with glutamate receptors. In addition to protein complex identification, we used TAP-MS to identify Kv4.2 phosphorylation sites. Several known and unknown phosphorylation sites were identified. These findings provide a novel path to identify protein-protein interactions and PTMs in neurons and shed light on mechanisms of neuronal signaling potentially involved in the pathology of neurological diseases.

## Introduction

Most proteins exert their function as part of a protein complex or “cellular machine” (Alberts, 1998; Gavin et al., 2002; Krogan et al., 2006). Characterization of these machines, building blocks of complex organization units such as pathways, is thought to be critical for the understanding of disease and represents a comprehensive approach toward the identification of new drug targets (Brown and Superti-Furga, 2003; Fishman and Porter, 2005; Burckstummer et al., 2006). Tandem affinity purification (TAP) is a generic two-step affinity purification protocol for isolation of TAP-tagged proteins together with their associated proteins. Compared with single-step immunoprecipitation, TAP can dramatically reduce the level of background proteins in the purified sample and has been used in previous studies (Gregan et al., 2007; Li, 2011; Carneiro et al., 2016; Seo et al., 2021). However, TAP protocols have to date only been applied in cell lines, but not for highly differentiated cells such as neurons.

Synapses are key neuronal structures in the brain. They are responsible for the transmission, integration, and storage of information in neurons. The synapse can be considered one of the most complex cellular organelles, consisting of thousands of proteins that interact in an activity-dependent manner (Biesemann et al., 2014; Taoufiq et al., 2020; Faust et al., 2021). Thus, the study of synaptic protein complex components is essential to unravel the molecular nature of the neuronal function.

**FIGURE 1:**
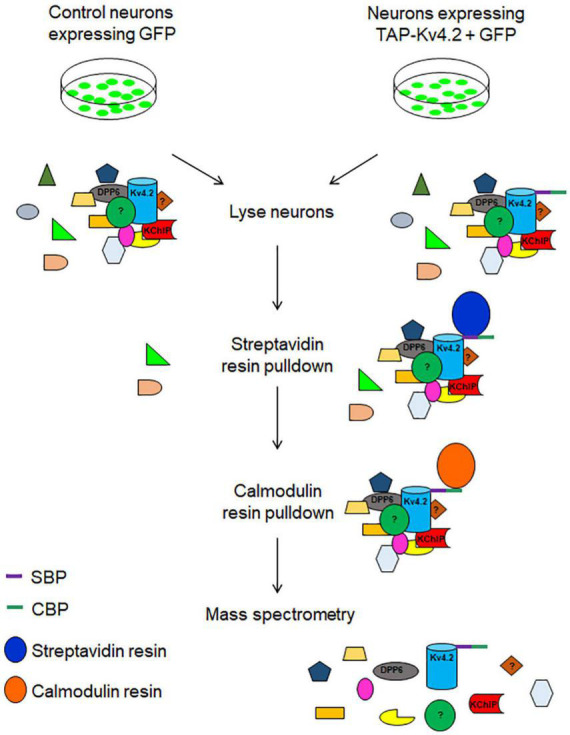
Schematic diagram of tandem affinity purification (TAP) of tagged Kv4.2 and interacting proteins using streptavidin resin followed by calmodulin resin. Hippocampal neurons expressing GFP or TAP-Kv4.2 plus GFP were lysed and subjected to streptavidin resin pulldown. After elution, samples underwent calmodulin resin pulldown. Pure Kv4.2 complex was isolated from neurons that express TAP-Kv4.2, while in control neurons, background proteins were removed. Purified samples were analyzed for mass spectrometry. DPP6 and KChIP are known binding proteins of Kv4.2.

Here, we demonstrate the use of TAP in neurons to identify protein complex constituents using Kv4.2 as an example. We identified new interacting proteins, including synaptic proteins (Table 1). As a member of the Shal-type family, Kv4.2 is the prominent A-type voltage-gated potassium channel expressed in hippocampal CA1 pyramidal neuron dendrites (Hoffman et al., 1997). Kv4.2 controls dendritic excitability, impacts neuronal plasticity, and contributes to learning and memory (Hoffman et al., 1997; Chen et al., 2006; Lugo et al., 2012; Hu et al., 2020b). Aberrant Kv4.2 function is also implicated in autism spectrum disorder (ASD) (Guglielmi et al., 2015), temporal lobe epilepsy (Bernard et al., 2004; Singh et al., 2006; Hall et al., 2015), and Fragile X syndrome (Gross et al., 2011; Lee et al., 2011). Therefore, it is important to study Kv4.2 synaptic protein complex elements that regulate its function. In our protocol, TAP-Kv4.2 was expressed in cultured hippocampal neurons by lentivirus. Neurons then were lysed and underwent streptavidin resin pulldown. After elution, samples were subjected to calmodulin resin pulldown. The samples with two-step purification were analyzed by mass spectrometry (Figure 1).

**TABLE 1:** Candidate proteins that were identified by TAP-MS in hippocampal neurons.

Weak lysis buffer			Weak lysis buffer			Strong lysis buffer			Strong lysis buffer		
High molecular weight proteins			Low molecular weight proteins			High molecular weight proteins			Low molecular weight proteins		
Unique peptides	Total peptides	Gene symbol	Unique peptides	Total peptides	Gene symbol	Unique peptides	Total peptides	Gene symbol	Unique peptides	Total peptides	Gene symbol
94	99	Dync1h1	44	112	Tubb2a	122	133	Dync1h1	13	15	Rps4x
90	110	Acaca	30	36	Atp5a1	**96**	**386**	**Kcnd2**	11	17	Rps18
56	59	Sptan1	28	59	Tuba1a	85	89	Sptan1	11	16	Rps3
42	125	Hspa8	25	43	Tubb3	52	56	Acaca	10	12	Tubb2a
40	47	Hspa5	24	29	Pccb	46	55	Sptbn2	10	11	Rpl7
39	40	Sptbn1	24	27	Vim	42	42	Sptbn1	9	22	Calm1
35	42	Pc	24	26	Mccc2	38	70	Atp2b1	9	12	Rps19
32	49	Tubb2a	21	24	Camk2a	37	40	Myo5a	**8**	**20**	**Kcnip1**
**30**	**40**	**Dpp6**	20	21	Gfap	34	56	Map6	8	17	Rala
28	31	Atp2b1	18	20	Rps3	32	43	Kab	**8**	**16**	**Kcnip4**
28	28	Mccc1	17	17	Atp5b	27	31	Ppfia3	8	8	Slc25a4
**27**	**126**	**Kcnd2**	15	16	Hspa8	26	30	Prrc2a	7	11	Atp5o
**26**	**26**	**Dpp10**	14	17	Ckm	26	28	Nos1	7	10	Ppp3ca
25	27	Iqsec2	14	14	Ppp3ca	**25**	**33**	**Kcnd3**	7	9	Tuba1a
25	25	Ubr4	13	16	Eef1a1	23	25	Sbf1	7	9	Rac1
24	32	Map6	13	15	Farsa	23	24	Ccdc88a	7	9	Hsd17b12
24	25	Srcin1	13	13	Bag5	22	24	Lrpprc	**7**	**8**	**Kcnip2**
21	22	Pcca	12	32	Alb	22	23	Srcin1	6	10	Rps7
20	21	Hnrnpu	**12**	**16**	**Kcnip4**	**21**	**35**	**Kcnd1**	6	8	Calml3
20	20	Sptbn2	12	15	Eno3	19	19	Kif21b	6	7	Rps16
19	32	Hspa2	12	13	Psmd3	19	19	Ubr4	6	6	Atp6v1d
18	39	Hspa9	12	13	Rps4x	19	19	Copa	6	6	Atp5c1
18	22	Syn1	12	12	Gapdh	17	18	Ank3	6	6	Slc25a5
18	18	Pfkm	11	27	Acta2	17	18	Gprin1	6	6	Rps9
17	18	Atp1a1	11	17	Rps19	16	16	Map1b	6	6	Rpl10a
17	17	Mccc2	11	12	Aldoa	15	22	Tubb2a	6	6	Rps3a
17	17	Psmd2	11	11	Rps7	15	17	Pcca	6	6	Nipsnap1
17	17	Prrc2a	10	13	Bag2	15	17	Prrc2c	6	6	Rpl13a
16	39	Tuba1a	10	11	Rala	15	16	Shank1	5	10	Bag2
16	16	Pccb	10	11	Psmc5	15	16	Unc13a	5	8	Rps10
16	16	Tenm2	10	10	Atp6v1d	15	15	Ckap5	5	8	Rps14
**15**	**17**	**Kcnd3**	10	10	Slc25a4	14	14	Ylpm1	5	8	Hist1h4b
15	17	Stxbp1	9	12	Camk2b	14	14	Itpr1	5	8	Rasl2-9
15	17	Eif4g2	9	10	Pkm	13	15	Clasp2	5	7	Slc25a1
15	16	Hnrnpm	9	10	Hadhb	13	14	Ubr5	5	7	Rps13
15	15	Ppp3ca	9	10	Atp5c1	13	13	Golga3	5	6	Uqcrb
15	15	Myo5a	9	9	Psmc1	13	13	Atp2b2	5	6	Sfxn3
14	15	Bag6	**9**	**9**	**Kcnip1**	13	13	Usp9x	5	6	Rps2
14	15	Prrc2c	8	11	Dcx	12	15	Caskin1	5	6	Rpl10
14	14	Map1b	8	9	Ppp2r2a	12	13	Agap2	5	6	Bdh1
14	14	Copa	8	9	Psmc4	12	12	Arhgap21	5	6	Rpl26
13	17	Tubb3	8	9	Phb2	12	12	Tenm2	5	5	Rps15a
13	16	Atp2b2	8	9	Rbm4	11	13	Atp2b4	5	5	Tfam
13	13	Pfkl	8	8	Slc1a3	11	12	Hnrnpu	5	5	Rpl13
13	13	Clasp2	8	8	Fasn	11	12	Pc	5	5	Rpl8
13	13	Ppfia3	8	8	Rps3a	11	12	Arhgap32	5	5	Rpl7a
13	13	Kpnb1	8	8	Rps18	11	11	Ankrd17	5	5	Tecr
13	13	Ddx5	7	9	Tubb5	11	11	Dock7	5	5	Mrps15
12	19	Camk2b	7	9	Slc25a3	11	11	Eif3a	**4**	**9**	**Kcnip3**
12	14	Kab	7	8	Actb	10	11	Hspa8	4	6	Gbas
12	13	Atp5a1	7	8	Ppp3cb	10	11	Myh10	4	5	Rab18
12	13	Ap3b2	7	8	Ina	10	10	Ap3b2	4	5	Stmn3
12	12	Mthfd1l	7	8	Calm1	10	10	Ehbp1	4	5	Rps25
12	12	Cand1	7	7	Syn2	10	10	Arhgap33	4	5	Rps8
12	12	Camk2a	7	7	Ca3	9	10	Tnrc6b	4	5	Arf1
11	13	Ccdc88a	6	67	Hbb	9	10	Rims1	4	4	Rps6
11	13	Usp9x	6	8	Hpx	9	10	Ppfia2	4	4	Stmn2
11	12	Sbf1	6	7	Hspd1	9	10	Arid1a	4	4	Rpl11
11	12	Usp20	6	7	Idh3B	9	9	Kif5c	4	4	Rpl18
11	11	Crmp1	6	7	Crmp1	9	9	Ctnnd1	4	4	Phb2
11	11	Hspd1	**6**	**7**	**Kcnip3**	9	9	Sipa1l1	4	4	Mrps23
11	11	Ina	6	7	Prdx2	9	9	Map2	4	4	Atp5f1
11	11	Dpysl2	6	7	Eif4g2	9	9	Tjp2	4	4	Slc25a11
11	11	Nos1	6	6	Rpl9	9	9	Ddx42	4	4	Diras2
10	11	Tnrc6b	6	6	Psmc3	8	10	Cyfip1	4	4	Phb
10	11	Wdr7	6	6	Phb	8	9	Mybbp1a	4	4	Map6
10	10	Atp1a3	6	6	Atp5o	8	9	Supt6h	4	4	Rpl23a
10	10	Acsbg1	6	6	Rpl7	8	9	Cand1	4	4	Hist1h1e
10	10	Map2	6	6	Pygm	8	8	Smarcc2	4	4	Ywhae
10	10	Kif21b	6	6	C3	8	8	Pikfyve	4	4	Ndufs4
10	10	Atp2a2	6	6	Rps13	8	8	Grin2b	4	4	Rps11
10	10	Matr3	5	6	Map6	8	8	Grm5	3	14	Taf8
10	10	Opa1	5	6	Brsk1	8	8	Dhx30	3	9	Stmn1
10	10	Pfkp	5	5	Stmn2	8	8	Arhgap23	3	5	Cyc1
9	23	Hspa1l	5	5	Stoml2	8	8	Dapk1	3	4	Rps20
9	12	Hsph1	5	5	Alg2	7	9	Wdr7	3	4	Slc25a18
9	10	Slc1a3	5	5	Mdh1	7	8	Tuba1a	3	4	Pcsk1n
9	10	Mogs	5	5	Rps10	7	8	Ank2	3	4	Rpl23
9	10	Dhx9	5	5	Pvalb	7	8	Plxna3	3	4	Kras
9	9	Ipo9	5	5	Ca1	7	8	Ccdc88a	3	4	Mpc2
9	9	Ipo7	5	5	Rps15a	7	8	Dpp6	3	3	Rpl19
9	9	Ddx1	5	5	Tf	7	8	Smarca4	3	3	Ppp3cb
8	9	Gcn1l1	5	5	A1m	7	8	Apc	3	3	Dhrs7b
8	9	Klc2	**5**	**5**	**Kcnip2**	7	7	Sugp2	3	3	Ndufs3
8	8	Hadha	4	6	Rac1	7	7	Sptbn4	3	3	Atp5i
8	8	Atp6v1a	4	5	Hsd17b12	7	7	Map1a	3	3	Sfxn1
8	8	Nsf	4	5	Gdap1	7	7	Camk2a	3	3	Atp5a1
8	8	Psmd3	4	5	Camk2g	7	7	Myo18a	3	3	Rps24
8	8	Lppr4	4	5	Tubb4b	7	7	Cacna1e	3	3	Rab33a
8	8	Rpn1	4	5	Cyc1	7	7	Rbm6	3	3	Arf6
8	8	Ank3	4	5	Tkt	7	7	Odz3	3	3	Rpl24
8	8	Atp2a1	4	5	Tpi1	7	7	Herc2	3	3	Rps23
8	8	Atad3	4	4	Hnrnpa2b1	6	7	Syngap1	3	3	Mylk2
**7**	**11**	**Kcnd1**	4	4	Dnaja1	6	7	Prpf8	3	3	Acp1
7	8	Hsp90ab1	4	4	Psmc2	6	6	Ap3d1	3	3	Rpl30
7	8	Unc13a	4	4	Bdh1	6	6	Ylpm1	3	3	Ptpmt1
7	8	Arhgap33	4	4	Vat1	6	6	Ppfia4	3	3	Rps5
7	7	Hspa1a	4	4	Rps6	6	6	Gapvd1	3	3	Rab5a
7	7	Dclk1	4	4	Rps14	6	6	Dhx9	3	3	Rpl27
7	7	Syn2	4	4	Eno1	6	6	Matr3	3	3	Vdac2
7	7	Ppp3cb	4	4	Acad9	6	6	Map7d1	3	3	Vdac3
7	7	Psmd1	4	4	Hspa2	6	6	Fam120a	3	3	Ndufa9
7	7	Shank1	4	4	Slc25a1	6	6	Ank3			
7	7	Acsl4	4	4	Gpd1l	6	6	Eif4g3			
7	7	Ndufs1	4	4	Prdx1	6	6	Myo6			
7	7	Grin2b	4	4	Uba52	6	6	Ascc3l1			
7	7	Atp2c1	4	4	Gsn	6	6	Sf3b2			
6	7	Brsk1	4	4	Sccpdh	6	6	Myo9a			
6	6	Hnrnpul2	4	4	Rpl4	5	7	Lrrc7			
6	6	Marcks	3	4	Hnrnpa1	5	6	Eprs			
6	6	Ranbp2	3	4	Rps8	5	6	Nrxn2			
6	6	Nefl	3	4	Serpina3n	5	6	Sf3b1			
6	6	Ckap4	3	4	Tardbp	5	6	Eif4g1			
6	6	Kif5c	3	4	Rps17	5	6	Uba52			
6	6	Taok1	3	4	Pgam2	5	6	Iqsec2			
6	6	Trim33	3	4	Ca2	5	6	Iqgap1			
6	6	Tjp2	3	4	Fam164a	5	6	Sipa1l3			
6	6	Dnajc16	3	4	Ces1c	5	5	Cltc			
6	6	Nckap1	3	4	Ppp2r1a	5	5	Akap12			
6	6	Pabpc1	3	3	Hnrnph1	5	5	Tjp1			
6	6	Camkv	3	3	Zwint	5	5	Prrc2b			
6	6	Soga3	3	3	Capza2	5	5	Tubb3			
6	6	Myef2	3	3	Arl10	5	5	Nefm			
6	6	Akap12	3	3	Lppr4	5	5	Safb			
6	6	Hsp90aa1	3	3	Ndufs3	5	5	Pkp4			
6	6	Sv2a	3	3	Cat	5	5	Dock7			
6	6	Kidins220	3	3	Mest	5	5	Thrap3			
6	6	Cyfip1	3	3	Tubb6	5	5	Xrn2			
6	6	Trim67	3	3	Eef1g	5	5	Rpl6			
6	6	Rbm4	3	3	Slc25a5	5	5	Grin1			
5	7	Micu1	3	3	Clu	5	5	Trip12			
5	5	Helz	3	3	Arf1	5	5	Fmnl2			
5	5	Copb1	3	3	Map2	5	5	Soga1			
5	5	Hspa4	3	3	Rpl23a	4	6	Mylk2			
5	5	Farsa	3	3	Dnajb12	4	5	Tsc2			
5	5	Atp6v0a1	3	3	Cfl1	4	5	Tanc2			
5	5	Hspa12a	3	3	Rplp0	4	5	Nckap1			
5	5	Slc27a1	3	3	Rpl10a	4	5	Opa1			
5	5	Dnajc10	3	3	Dpysl2	4	5	Nup153			
5	5	Cct3	3	3	Fabp4	4	4	Hsp90aa1			
5	5	Ctnnb1	3	3	Rpl11	4	4	Ttbk1			
5	5	Mtor	3	3	Map1b	4	4	Ank2			
5	5	Atp2b4	3	3	Pgk1	4	4	Mccc1			
5	5	Dlat	3	3	Fn1	4	4	Camk2b			
5	5	Wdr48	3	3	Nipsnap1	4	4	Zfr			
5	5	Syt1	3	3	Stmn3	4	4	Fasn			
5	5	Vim	3	3	Rps2	4	4	Map4			
5	5	Dpysl5	3	3	Slc4a1	4	4	Scn2a			
5	5	Eif2c2	3	3	Pc	4	4	Ctnnd2			
5	5	Cherp	3	3	Dnaja2	4	4	Ascc3			
4	7	Uba52	3	3	Rasl2-9	4	4	Mthfd1l			
4	6	Abcd3	3	3	Elavl2	4	4	Cherp			
4	5	Ddb1	3	3	Ldha	4	4	U2surp			
4	5	Brsk2	3	3	Agk	4	4	Tpr			
4	5	Cpeb4	3	3	Ddx5	4	4	Tanc2			
4	5	Cpeb2	3	3	Pygb	4	4	Rasgrf2			
4	5	Ncl	3	3	Dnaja3	4	4	Chd4			
4	5	Cltc	3	3	Rps11	4	4	Iars			
4	4	Pfkm	3	3	Rpl3	4	4	Mast1			
4	4	Rph3a	3	3	Rpl13a	4	4	Myo1b			
4	4	Frmd4a				4	4	Pi4ka			
4	4	Tubb5				4	4	Hdac4			
4	4	Dpysl3				4	4	Flna			
4	4	Camk2g				3	10	Taf8			
4	4	Ubr5				3	4	Sptbn1			
4	4	Ckap5				3	4	Ube3c			
4	4	Kif5b				3	4	Ppp3ca			
4	4	Cacna1e				3	4	Uhrf1bp1			
4	4	Gpd2				3	3	Atp2b3			
4	4	Trim3				3	3	Camsap2			
4	4	Slc25a13				3	3	Sart1			
4	4	Xrn2				3	3	Kif1b			
4	4	Pnpla8				3	3	Rpl18			
4	4	Dock7				3	3	Rptor			
4	4	Osbpl6				3	3	Hspa4			
4	4	Lrrc7				3	3	Calm1			
4	4	Adcy1				3	3	Rims2			
4	4	Acad9				3	3	Shank2			
4	4	Numb				3	3	Hspa5			
4	4	FAM120C				3	3	Wnk2			
4	4	Ank2				3	3	Shank3			
4	4	Amfr				3	3	Smarca2			
4	4	Syn3				3	3	Abl2			
4	4	Dhx15				3	3	Lppr4			
4	4	Arhgap32				3	3	Ipo5			
4	4	Nefm				3	3	Flii			
4	4	Grin1				3	3	Crebbp			
4	4	Abce1				3	3	Epha4			
4	4	Mark1				3	3	Brsk1			
4	4	U2surp				3	3	Taf10			
4	4	Vac14				3	3	Ikbkap			
4	4	Ddx42				3	3	FAM120C			
4	4	Ccdc88a				3	3	Nav3			
4	4	Bsn				3	3	Acta2			
3	4	Dync1li1				3	3	Dctn1			
3	4	Gigyf1				3	3	Pnpla6			
3	4	Numbl				3	3	Mtor			
3	4	Rhot1				3	3	Lars			
3	4	Tex10				3	3	Ccdc88c			
3	3	Ptcd3				3	3	Nrxn1			
3	3	Kif5a				3	3	Dlgap4			
3	3	Myo6				3	3	Dpysl2			
3	3	Osbpl8				3	3	Nf1			
3	3	Atp2b3				3	3	Kif5b			
3	3	Ncdn				3	3	Cacna1b			
3	3	Eef1a1				3	3	Rpl4			
3	3	Ipo5				3	3	Pfkm			
3	3	Fus				3	3	Scn1a			
3	3	Osbpl11				3	3	Tanc1			
3	3	Usp7				3	3	Pum1			
3	3	Ank2				3	3	Pex1			
3	3	Bag5				3	3	Farp1			
3	3	Lrpprc									
3	3	Pccb									
3	3	Canx									
3	3	Smarcc2									
3	3	Diaph1									
3	3	Grm5									
3	3	Ankrd17									
3	3	Hspa4l									
3	3	Madd									
3	3	Agap2									
3	3	Dctn1									
3	3	Ctnnd2									
3	3	Dclk2									
3	3	Gprin1									
3	3	Kif2a									
3	3	Mta2									
3	3	Wfs1									
3	3	Tnrc6b									
3	3	Shank2									
3	3	Kif3a									
3	3	Mta1									
3	3	Ctnnd1									
3	3	Slc27a4									
3	3	Tomm70a									
3	3	Prkca									
3	3	Mark2									
3	3	Plxna3									
3	3	Dync1h1									
3	3	Atp2b4									
3	3	Tcp1									
3	3	Mybbp1a									
3	3	Ppp2r1a									
3	3	Atp12a									
3	3	Mtmr1									
3	3	Sf3b2									

The numbers of unique and total peptides are shown for each protein identified by TAP-MS in hippocampal neurons with both weak lysis buffer (0.1% NP40) and strong lysis buffer (1% Triton X-100 and 0.5% deoxycholate). The number of total peptides indicates the abundance of a protein in the sample, the larger the number, the higher abundance of the protein. The number of unique peptides shows the number of peptide hits by removing the redundancy peptides. It determines the sequence coverage of corresponding proteins and the confidence of protein identification, the larger the number, the higher the sequence coverage and confidence of the protein. Kv4, DPP, and KChIP families are shown in bold.

Post-translational modifications (PTMs) have a strong impact on proteins across all kingdoms of life, affecting multiple functional and chemical properties of their protein recipients. Dysregulations in PTMs have been implicated in various dysfunctions and diseases (Clark et al., 2022; Yoo et al., 2022). The expression and modification of synaptic proteins are dynamically regulated, depending on the neuronal activity, which underlies synaptic plasticity (Malenka and Bear, 2004). Glutamate receptors such as AMPA receptors (GluR) and NMDA receptors (GluN) undergo trafficking and altered sub-cellular localization upon phosphorylation (Lee et al., 2000; Lee, 2006). Voltage-gated ion channels including Kv4.2 are also regulated by phosphorylation (Park et al., 2008; Shipston and Tian, 2016; Montenarh and Gotz, 2020; Li et al., 2021). Kv4.2 phosphorylation at S552 is required for activity-dependent Kv4.2 channel trafficking (Lin et al., 2010). Kv4.2 phosphorylation at T602 and T607 allows Pin1 binding leading to isomerization of Kv4.2 impacting cognitive flexibility (Hu et al., 2020b). Using the similar protocol described above, we identified Kv4.2 core phosphorylation sites as well as new candidate sites. Taken together, this TAP plus lentivirus protocol provides a power tool to identify protein complex and PTMs in primary neurons.

## Materials and methods

### Reagents

OPTI-MEM (Gibco 31985), Chloroquine (Sigma C6628), 0.45 μm low protein binding filter (Corning 430768), Opti-prep density gradient medium (Sigma D1556-250 mL), Poly-L-Lysine (Sigma P-2636), Trizma buffer pH 8.5 0.1 M (Sigma T1194), B27 supplements (Gibco 17504044), Papain (Worthington LS003119), Characterized Fetal Bovine Serum (Hyclone SH30071.03), 10 × HBSS (Gibco 14185-052), Pen/strep (Gibco 15140122), Pyruvate (Gibco 11360070), Hepes (Gibco 15630080), Glucose (Sigma G8270), Ara C (Sigma C-6645), DNase (Sigma DN-25), Neurobasal Media (Gibco 21103-049), Triton X-100 (Sigma T8787), Deoxycholate (Sigma D6750), Sample loading buffer (Invitrogen NP0007), Sample reducing agent (Invitrogen NP0009), phosSTOP (Roche, 04906837001), Complete EDTA–Free protease inhibitors (Roche 56079200), Silver staining (Invitrogen LC6070), and Coomassie staining (LC6060).

### Expression constructs and subcloning

The human Myc-DDK-Kv4.2 construct was purchased from Origene (RC215266). Kv4.2 was subcloned into the TAP tag vector that was obtained from Agilent (pCTAP, #240102). C-terminus TAP-tagged Kv4.2 was then subcloned into the lentivirus vector (modified FUWIG from Dr. Paul Worley’s lab) to generate TAP-Kv4.2 lentivirus vector. All constructs were verified by sequencing.

### Lentivirus generation

Lentivirus was generated using HEK293FT cells, which were cultured with DMEM medium with 10% FBS.

Day 1: (1) morning—Coat 175T flask with 10 mL poly-L-lysine (PLL, 0.1 mg/mL) for 3 h at 37°C, wash with PBS 3 times, (2) afternoon—Split 293FT cells and plate 1.8 × 10^7^ cells/175 cm^2^ flask.

Day 2: (1) 9:00 a.m.—Remove medium, add 20 mL serum-free OPTI-MEM with GlutaMax and 25 μM chloroquine, (2) 11:00 a.m.—Transfection: (a) Mix 2 mL OPTI-MEM with 70 μL X-tremeGENE HP for each flask, RT 5 min, (b) Add DNA—15 μg pFUWIG or pFUWIG-Kv4.2-TAP + 20 μg pΔ8.9 + 10 μg pVSVG, RT 15 min, add to medium, (3) 6:00 p.m.—Supplement with 10 μM Sodium Butyrate (boosting of lentiviral transduction).

Day 3: 9:00 a.m.—Discard media and replace with 20 mL serum-free OPTI-MEM with GlutaMax.

Day 4: 9:00 a.m.—(1) Collect medium (containing lentivirus), add new 20 mL serum-free OPTI-MEM with GlutaMax, (2) Centrifuge collected medium (2,000 g) for 10 min at 4°C, store supernatant at 4°C.

Day 5: 9:00 a.m.—(1) Collect medium (containing lentivirus), 2,000 g centrifuge for 10 min at 4°C, (2) Filter supernatant with 0.45 μm low protein binding filter, (3) Concentration: (a) Add 100 μL Opti-prep density gradient medium (Sigma) to Beckman tubes, (b) Add ∼ 38 mL supernatant, (c) 25,000 rpm for 2 h at 4°C, (d) Remove 37 mL medium and discard. Mix, aliquot, and store the remaining 1.1 mL at 4°C. (e) Infect HEK293T cells with a series of dilution (1:10,000; 1:3,000; 1:1,000; 1:300; 1:100) to examine the virus titer.

### Neuronal culture and lentivirus infection

Rat hippocampal neurons were cultured from embryonic day 18 (E18) pups. Dissection and plating procedures were as follows.

1. 10 cm dishes were coated with 0.5 mg/mL poly–L–lysine and incubated at 37°C overnight.
2. Preparation. (a) 10 cm plates with ice-cold dissection medium (DM) for use as dissection dishes, (b) 15 mL conical with 2 mL ice-cold DM, (c) sterilize tools in 70% EtOH, and (d) pre-warm papain and DNAase to 37°C.
3. Dissection. (a) After euthanasia of the mother rat, remove pups, (b) remove pup brains to fresh dish with ice cold DM immediately after removal from skull, (c) remove hemispheres and then meninges by sliding tweezers in hole left by olfactory bulb, and (d) peel hippocampus away from cortex.
4. Add 67 μL of papain and 20 μL of DNase (final concentration of 0.01%) to 2 mL of dissection media. Incubate in water bath (37°C) with gentle perturbation every 5 min for 20 min.
5. Warm 50 mL of NM5 to 37°C and thaw B27.
6. Prepare dissociation pipets. Use two or three fire-polished Pasteur pipets with sequentially smaller tip diameters.
7. Aspirate the solution and add 2 mL of pre-warmed NM5 with freshly added B27. Wash 1X with the NM5 and remove, and then add 2 mL of NM5.
8. Dissociate the tissue by gently triturating the hippocampi through a fire-polished Pasteur pipette. Starting with the largest pipet, gently triturate 5–6X, shooting tissue against the wall of the tube to avoid bubble formation. Remove supernatant to a fresh tube, gently add 2 mL NM5 and triturate with a smaller pipet.
9. Dilute the cell mixture to 10 mL with plating media and then run through a 70 μM cell strainer.
10. Spin cells down at 1,000 rpm for 5 min.
11. Re-suspend cells in NM5 + B27.
12. Dilute cell stocks 1:2 in Trypan blue. Count with a hemocytometer. Only count bright cells. Don’t forget to factor in dilution for final cell concentration. Around 1.0 × 10^6^ neurons per rat pup should be obtained.
13. Aliquot enough neurons into a sterile tube (5 × 10^6^ cells for each 100 mm dish), add FUWIG-Kv4.2-TAP or control Lentivirus, and incubate at 37°C for 1 h with gentle mixing every 25 min.
14. Wash the poly–L–lysine-coated dishes three times with PBS. Add 5 mL NM5 to each dish and place them at 37°C.
15. Plate 5 × 10^6^ cells for each 10 cm dish and incubate at 37°C.
16. Change media to fresh NM5 after 1–2 h. Cells should be attached to the plates.
17. Check cells daily. On DIV3 replace 1/2 media (remove 4.5 mL, add 5 mL) with glia-conditioned NM1 (add B27 and AraC right before feeding glia-conditioned NM1 to Neurons).
18. Cultures are then fed with conditioned NM1 + B27 by 1/2 media changes every 3rd day (remove 4.5 mL and add 5 mL) to protect against media evaporation and metabolic byproduct accumulation. For DIV 3 and older, neurons should be fed with NM1 that has been Glial conditioned overnight.

### Tandem affinity purification-mass spectrometry assay

Briefly, excised gel bands were cut into ∼1 mm^3^ pieces. The gel pieces were then subjected to in-gel trypsin digestion and dried. Samples were reconstituted in 5 μL of HPLC solvent A (2.5% acetonitrile, 0.1% formic acid). A nano-scale reverse-phase HPLC capillary column was created by packing 2.6 μm C18 spherical silica beads into a fused silica capillary (100 μm inner diameter × ∼30 cm length) with a flame-drawn tip. After equilibrating the column, each sample was loaded *via* a Famos autosampler (LC Packings, San Francisco, CA). A gradient was formed and peptides were eluted with increasing concentrations of solvent B (97.5% acetonitrile, 0.1% formic acid). As peptides eluted, they were subjected to electrospray ionization and entered into an LTQ Velos ion-trap mass spectrometer (Thermo Fisher Scientific, Waltham, MA). Peptides were detected, isolated, and fragmented to produce a tandem mass spectrum of specific fragment ions for each peptide. Peptide sequences (and hence protein identities) were determined by matching protein database^1^ with the acquired fragmentation patterns using software program, SEQUEST (Thermo Fisher Scientific, Waltham, MA). All the data was filtered to less than two percent peptide false discovery rate and less than five percent protein false discovery rate.

Hippocampal neurons were harvested at DIV14. TAP-Kv4.2 was purified using the TAP purification kit from Agilent (#240107) with some modifications. We use the kit provided lysis buffer that contains 0.1% NP-40 or stronger lysis buffer (1% Triton X-100, 0.5% deoxycholate in PBS, pH 7.4) to lyse hippocampal neurons. Lysis buffers were supplemented with phosSTOP and Complete™ EDTA–Free protease inhibitors before lysis. 1 mL lysis buffer is used for each 10 cm dish. Samples underwent streptavidin resin and then calmodulin resin two-step purification according to manufactory’s protocol. Purified samples were eluted by sample loading buffer and separated by SDS-PAGE on a 10% NuPAGE gel (Novex/Invitrogen). To visualize protein bands, silver staining (Invitrogen LC6070) and Coomassie staining (LC6060) were performed according to manufactory’s instructions. The gel bands or fragments were excised and sent to the Taplin Mass Spectrometry Facility at Harvard University for in-gel digestion using trypsin and mass spectrometric analysis.

Ascore was used for phospho-sites identification (Beausoleil et al., 2006). There were two Ascores produced at two different fragmentation ion tolerances for each peptide. If the Ascore1a and Ascore1b are both higher than 13 the site shown in the Ascore Seq column is considered confidently assigned. If there are two or more sites in a phosphuretted peptide, Ascore1 refers to the N-terminal most site and Ascore2 the next site moving toward the C-terminal.

### Antibodies

Mouse anti-Kv4.2 (NeuroMab, 75-016) was used at 1:200 for immunostaining; rabbit anti-Kv4.2 (Sigma, HPA029068) was used at 1:200 for staining, 1:2,000 for western blot; mouse anti-GluN1 (NeuroMab, 75–272) was used at 1:100 for immunostaining; mouse anti-GluN2B (NeuroMab, 75–097) was used at 1:200 for immunostaining; rabbit anti-mGluR5 (Abcam, ab76316) was used at 1:100 for immunostaining; Myc (Millipore, 05–419) was used at 1:5,000 for western blot; Actin (Sigma, A-1978) was used at 1:10,000 for western blot; mouse anti-GFP (Invitrogen, A-11120) was used for IP; rabbit anti-GFP (Invitrogen, A-6455) was used at 1:2,000 for western blot; Alexa Fluor 488 goat anti-mouse (Invitrogen, A-11029) was used at 1:500; Alexa Fluor 488 goat anti-rabbit (Invitrogen, A-11034) was used at 1:500; Alexa Fluor 555 goat anti-mouse (Invitrogen, A-21424) was used at 1:500; Alexa Fluor 555 goat anti-rabbit (Invitrogen, A-21429) was used at 1:500; Alexa Fluor 680 goat anti-mouse (Invitrogen, A-21057) was used at 1:10,000; Alexa Fluor 680 goat anti-rabbit (Invitrogen, A-21076) was used at 1:10,000; IRDye 800CW goat anti-mouse (Licor, 926-32210) was used at 1:10,000, IRDye 800CW goat anti-rabbit (Licor, 926-32211) was used at 1:10,000.

### Cell culture and transfection for co-immunoprecipitation

HEK-293T cells used in biochemistry experiments were obtained from Dr. Paul Worley’s lab (Hu et al., 2012). HEK-293T cells were cultured in DMEM medium containing 10% FBS. Transfections were performed with X-tremeGENE 9 according to the manufacturer’s specifications. Cells were harvested about 40 h after transfection.

### Co–immunoprecipitation assays

HEK-293T cells were used in co–immunoprecipitation assays as previously reported (Hu et al., 2012). Briefly, 400 mL of IP buffer (1 X PBS, pH 7.4, with 1% Triton X–100, phosSTOP, and Complete™ EDTA–Free protease inhibitors) were added to the tissues and the samples were sonicated. After centrifugation, the supernatant containing 2 mg of proteins was mixed with 1–2 μg of anti-mouse GFP antibody for 3 h at 4°C. Next, 50 μL of 1:1 protein G–Sepharose slurry (GE Healthcare) or Magnetic beads was added for an additional 2 h. The protein beads were washed three times with IP buffer containing 1% Triton X–100. The protein samples were eluted with SDS loading buffer and analyzed by gel electrophoresis and western blotting.

### Western blot and quantification

Protein samples were mixed with 4x LDS sample buffer (Invitrogen NP0007) and 10 × sample reducing agent (Invitrogen NP0007) to a final concentration of 1 ×. Samples were loaded on 4–12% Bis-Tris gradient gel (Invitrogen 12-well, NP0322; 15-well, NP0323). The proteins were transferred to Immobilon-FL PVDF membrane (EMD Millipore, IPFL00010). The membrane was blocked with Odyssey blocking buffer (Li-COR, 927-40000) for 1?h at room temperature, followed by incubation with primary antibody in PBS overnight at 4°C. The membrane was then washed with PBST (PBS, pH 7.4, and 0.1% Tween-20) three times and incubated with secondary antibody in PBS for another hour. After three washes with PBS, the membrane was scanned using an Odyssey imaging system (LI-COR) according to the manufacturer’s protocol. Quantification of western blots was carried out using the gel analysis function in ImageJ within the linear range of detection which is determined by using serial dilutions of a representative sample.

### Immunostaining

Cultured hippocampal neurons (DIV14) were fixed with 4% paraformaldehyde (PFA) and permeabilized with 0.2% Triton X-100 in PBS. Cells were then blocked with 10% horse serum at RT for 1 h and then incubated with anti-glutamate receptor antibodies and Kv4.2 antibodies at 4°C overnight. After washing, cells were incubated with anti-mouse-555 and anti-rabbit-488 secondary antibodies at RT for 1?h. After washing, cells were then mounted on slides with anti-fade mounting medium containing 4’,6-diamidino-2-phenylindole (DAPI, Invitrogen, P36962) and imaged using a Zeiss 710 laser scanning confocal microscope equipped with a × 63 objective.

## Results

### Tandem affinity purification-tagged Kv4.2 lentivirus generation

**FIGURE 2:**
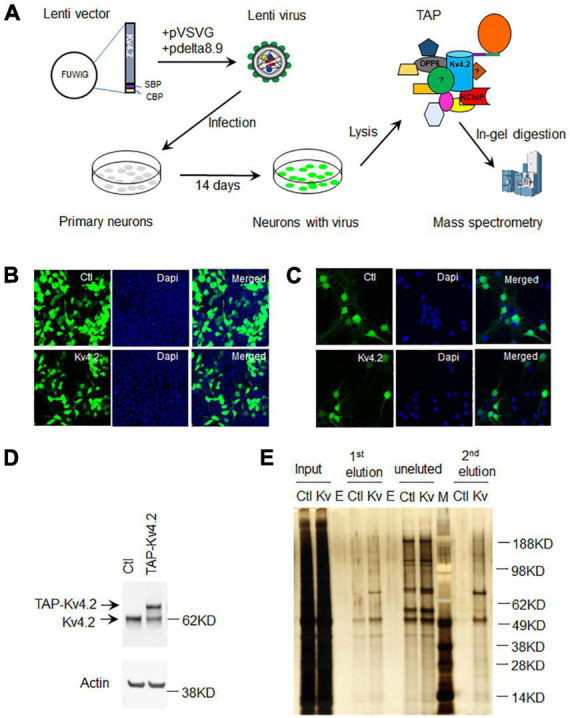
Purification of Kv4.2 protein complex using TAP in culture hippocampal neurons. **(A)** Schematic diagram of Kv4.2 protein complex purification using TAP in cultured hippocampal neurons. **(B)** Lentivirus expression of GFP or TAP-Kv4.2 plus GFP in HEK293FT cells (DIV 5). **(C)** Lentivirus expression of GFP or TAP-Kv4.2 plus GFP in hippocampal neurons (DIV10 after infection). **(D)** Western blot showing TAP-Kv4.2 expression in DIV14 hippocampal neurons. **(E)** Silver staining showing purified TAP-Kv4.2 protein complex in DIV14 hippocampal neurons. Note that there are plenty of non-specific proteins in control TAP purification after streptavidin resin pulldown (1st elution), while non-specific proteins are not visible after calmodulin resin pulldown (2nd elution), demonstrating the effectiveness of TAP in cultured hippocampal neurons. E, empty; M, marker.

We employed a lentiviral expression system to express TAP-Kv4.2 in cultured hippocampal neurons. To generate TAP-tagged Kv4.2 lentivirus, we first subcloned human Kv4.2 cDNA into CTAP vector to add TAP tags [streptavidin-binding-peptide (SBP) and calmodulin-binding-peptide (CBP)] to Kv4.2 C-terminus, and then subcloned TAP-tagged Kv4.2 into lentiviral vector FUWIG (Figure 2A). TAP-tagged Kv4.2 lentivirus was generated in HEK293FT cells with helper pVSVG and pdelta8.9. The virus expresses TAP-Kv4.2 and GFP since it contains an IRES-GFP element. HEK293T cell infection experiment showed that the control virus and TAP-Kv4.2 virus were generated successfully (Figure 2B).

### Hippocampal neuronal culture and lentivirus infection

Hippocampal neurons were cultured from E18 rat pups. Cells were infected with either control or TAP-Kv4.2 lentivirus on the same day of culture (Figure 2A). Lentivirus started to express the gene of interest after 3–4 days of infection. The expression gradually increased and was sustained until neurons were harvested at 2 weeks in culture (Figure 2C). TAP-Kv4.2 is reasonably well expressed after 2 weeks infection, comparable to endogenous Kv4.2 (Figure 2D).

### Tandem affinity purification of the Kv4.2 complex and mass spectrometry

Neurons were lysed at DIV14 with the lysis buffer provided by the InterPlay mammalian TAP purification kit and subjected to TAP purification (Figures 1, 2A). Silver staining showed that TAP-Kv4.2 and its complex were successfully pulled down and purified (Figure 2E). After the first step with streptavidin resin pull down, the eluted control sample contained significant amounts of non-specific proteins (Figure 2E). However, after the additional calmodulin resin pull-down, non-specific proteins were not detected in the eluted control sample (Figure 2E), suggesting the two-step TAP of Kv4.2 complex worked well and supporting the effectiveness of TAP.

Purified samples were separated by SDS-PAGE on a 10% NuPAGE gel. Quick blue staining was used to visualize TAP-Kv4.2 and its binding proteins. The gel was cut into two fragments that contained high molecular weight proteins and low molecular proteins, and then sent to the Taplin Mass Spectrometry Facility at Harvard University for in-gel digestion using trypsin and mass spectrometric analysis. Kv4.2 channels are known to function in macromolecular protein complexes with accessory subunits, including the K^+^ channel interacting proteins (KChIP1–4) and dipeptidyl peptidases 6 and 10 (DPP6 and DPP10) (Pongs and Schwarz, 2010; Kise et al., 2021). Successful Tandem affinity purification-mass spectrometry (TAP-MS) of the Kv4.2 complex should then identify DPP and KChIP family members. Indeed, the mass spec result showed that DPP6/10 and KChIP1-4 were among the list of interacting proteins (Table 1). Kv4 forms multimers with DPPs and KChIPs as demonstrated by the crystal structure (Pioletti et al., 2006; Wang et al., 2007; Kise et al., 2021). We found here that the other two Kv4 members, Kv4.1 and Kv4.3, are in the Kv4.2 complex (Table 1), which suggests that Kv4.2 can form heteromultimers with Kv4.1 and Kv4.3. Heteromultimers of Kv4 subunits with various DPPs and KChIPs combinations, may result in different channel expression and/or properties and function. Moreover, ribosomal proteins, proteasome 26S subunits, kinases, phosphatases and motor proteins are also identified (Table 1). In addition, we identified a number of synaptic receptors including GluN1, GluN2B, mGluR5, PlexinA3, and ion channels, e.g., Cav2.3 that we reported before (Murphy et al., 2022), and synaptic anchoring proteins, such as Shank1, Shank2 (Table 1).

The above TAP purification of Kv4.2 complex utilized kit provided lysis buffer which contains very mild detergent. To examine if this method works in strong lysis buffers so that higher affinity binding proteins of Kv4.2 can be isolated, we used 1% Triton X-100 with 0.5% sodium deoxycholate as lysis detergents. With the same purification procedure and mass spec, we showed again that DPP and KChIP family members are among the mass spec list of binding proteins (Table 1), suggesting the strong lysis buffer worked for purifying Kv4.2 complex. Kv4.1 and Kv4.3 were also pulled down by Kv4.2. In addition, most synaptic proteins that were identified using the mild lysis buffer were also found when using the strong lysis buffer (Table 1). These data suggest that TAP-MS of Kv4.2 complex in hippocampal neurons was successful and provided a novel method to identify interacting proteins in neurons.

### Validation of novel Kv4.2 binding partners identified by mass spectrometry

**FIGURE 3:**
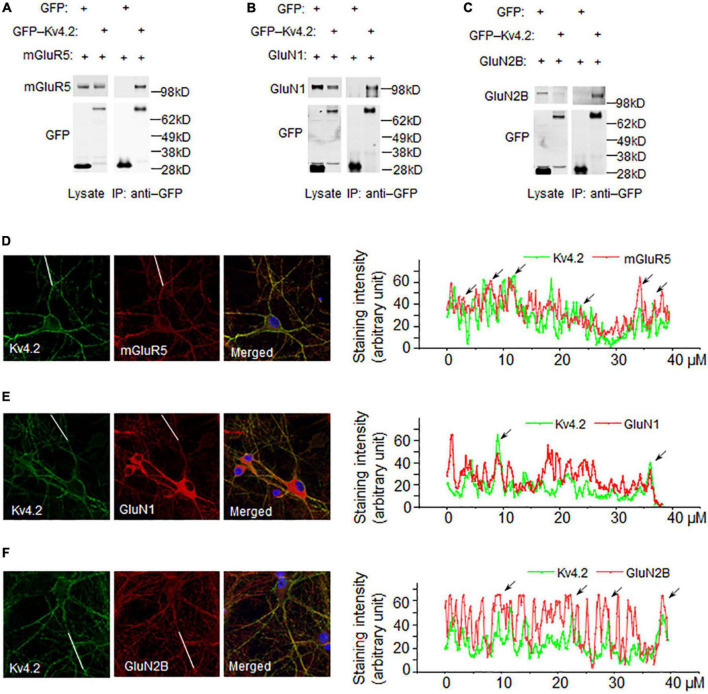
Co-immunoprecipitation and co-localization of Kv4.2 with novel interacting proteins. **(A)** mGluR5 binds to Kv4.2. GFP-Kv4.2 and HA-mGluR5 were co-transfected into HEK-293T cells. Detergent lysates were incubated with anti-GFP antibody and analyzed by western blotting with anti-GFP and anti-HA antibodies. **(B)** GluN1 binds to Kv4.2. GFP-Kv4.2 and HA-GluN1 were co-transfected into HEK-293T cells. Detergent lysates were incubated with anti-GFP antibody and analyzed by western blotting with anti-GFP and anti-GluN1 antibodies. **(C)** GluN2B binds to Kv4.2. GFP-Kv4.2 and HA-GluN2B were co-transfected into HEK-293T cells. Detergent lysates were incubated with anti-GFP antibody and analyzed by western blotting with anti-GFP and anti-HA antibodies. **(D)** mGluR5 colocalized with Kv4.2. mGluR5 and Kv4.2 were antibody-stained in cultured DIV14 hippocampal neurons. Line scan along the dendrites showed mGluR5 and Kv4.2 are colocalized as indicated with arrows. **(E)** GluN1 colocalized with Kv4.2. GluN1 and Kv4.2 were antibody-stained in cultured DIV14 hippocampal neurons. Line scan along the dendrites showed GluN1 and Kv4.2 are colocalized as indicated with arrows. **(F)** GluN2B colocalized with Kv4.2. GluN2B and Kv4.2 were antibody-stained in cultured DIV14 hippocampal neurons. Line scan along the dendrites showed GluN2B and Kv4.2 are colocalized as indicated with arrows.

Kv4.2 functions at synapses and dendrites with a gradient distribution along dendrites (Hoffman et al., 1997). Kv4.2 trafficking is regulated by NMDA receptor activation (Jung et al., 2008) and Kv4.2 regulates NMDAR subunit composition (Kim and Hoffman, 2012). The binding of Kv4.2 and NMDA receptors provides a mechanism for the interplay between Kv4.2 and NMDA receptors. Here, we examined if Kv4.2 binds to NMDA receptors when co-expressed in HEK293T cells. The results showed that both GluN1 and GluN2B co-immunoprecipitated with Kv4.2 (Figures 3A,B). In addition, we confirmed that mGluR5 co-immunoprecipitated with Kv4.2 when expressed in HEK293T cells (Figure 3C). Next, we examined if Kv4.2 colocalized with glutamate receptors in neurons. We co-stained Kv4.2 with GluN1, GluN2B, and mGluR5 in cultured hippocampal neurons and found that Kv4.2 colocalized with all three (Figures 3D–F).

### Identification of Kv4.2 phosphorylation sites

**FIGURE 4:**
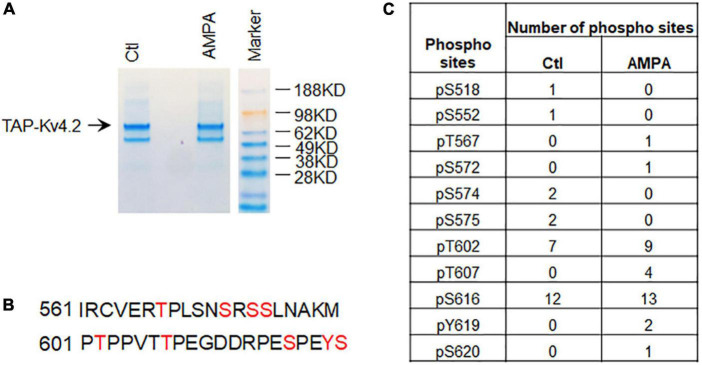
Identification of Kv4.2 phosphorylation sites in hippocampal neurons by TAP. **(A)** Coomassie staining showing TAP-Kv4.2 is purified by TAP using stronger lysis buffer (1% Triton X-100 and 0.5% DOC) in the control and AMPA treatment conditions. **(B)** Phospho-peptides of Kv4.2 that contain multiple phosphorylation sites identified by TAP-MS. **(C)** Number of phospho-sites in Kv4.2 were identified by TAP-MS in control and AMPA (50μM for 15 min) treatment in culture hippocampal neurons. Note that T607 phospho-peptides but not T602 and S616 phospho-peptides were increased in AMPA treatment compared to control.

Next, we examined if TAP-MS can be used for identification of modifications of Kv4.2 in cultured neurons. To purify TAP-Kv4.2, we used a strong lysis buffer to minimize Kv4.2 interacting proteins. TAP-Kv4.2 was purified using TAP protocol (Figure 4A). To examine if neuronal activity alters Kv4.2 phosphorylation, we treated DIV14 hippocampal neurons with 50μM AMPA for 15 min and subjected them to the same TAP-Kv4.2 purification as control (Figure 4A). SDS-page gels with TAP-Kv4.2 were excised and sent for mass spec using trypsin and chymotrypsin double digestion to identify Kv4.2 phosphorylation sites. The mass spec analysis identified a number of Kv4.2 phosphorylation sites (Figures 4B,C), including sites that have been previously reported, e.g., S552 (Hammond et al., 2008), T602 (Adams et al., 2000; Hu et al., 2020b), T607 (Adams et al., 2000; Hu et al., 2020b), and S616 (Hu et al., 2006). Interestingly, four T607 phosphorylation peptides were detected in AMPA treatment condition (83 total peptides, Supplementary Table 2), while no T607 phosphorylation peptide was detected in control condition (68 total peptides, Supplementary Table 1), suggesting that T607 phosphorylation may be triggered by activity in cultured neurons. By contrast, T602 phosphorylation and S616 phosphorylation are most abundant (7 and 12 phospho-peptides, respectively) and not altered by AMPA treatment (9 and 13 phospho-peptides, respectively) (Figure 4C). This is consistent with our previous reports that T607 phosphorylation but not T602 phosphorylation was induced by seizure and learning and memory tasks (Hu et al., 2020a,b). These data suggested that TAP-MS can be used for identification of modifications of neuronal proteins in cultured neurons.

## Discussion

The present study describes a protocol to identify protein complexes and PTMs in primary neurons. There are a few methodologies commonly used to examine protein–protein interaction: yeast two-hybrid (Y2H) screening (Fields and Song, 1989), proteomics analysis of immuno-precipitated protein complexes (Collins et al., 2006), and TAP-MS (Rigaut et al., 1999). Y2H screening examines the binary interaction of proteins. It may detect both strong and transient interactions. KChIPs were identified by Y2H (An et al., 2000; Nadal et al., 2003). However, Y2H does not provide information on the spatio-temporal pattern of the protein interactions. Immunoprecipitation (IP) pulls down the protein complexes using antibodies but is limited by the antibody specificity. DPPX was identified using anti-Kv4.2 antibodies crosslinked to protein-A Sepharose 4B beads in rat brain (Nadal et al., 2003). In another Kv4.2 antibody IP study, co-immunoprecipitation was not very successful when immunoprecipitating Kv4.2 using the NeuroMab antibody to isolate Kv4.2 protein complex (Klemmer et al., 2009). TAP protocol uses two-step affinity purification, dramatically reducing background proteins. We previously used TAP-Kv4.2 and identified Pin1 in HEK 293T cells (Hu et al., 2020b). In the present study, we employed lentiviral expression of TAP-Kv4.2 and identified Kv4.2 interacting proteins in cultured hippocampal neurons. Our data showed that there are no detectable proteins in the control TAP sample after the second elution, while after the first elution, there are plenty (Figure 2E). We didn’t include a MS analysis for the control sample, which might increase the possibility of false positive candidates. The level of lentiviral expression of TAP-Kv4.2 is similar to endogenous Kv4.2 so that it shouldn’t dramatically alter neuron status because of overexpression (Figure 2D). The lentiviral expression of TAP-Kv4.2 is also long lasting and doesn’t show toxicity to cultured hippocampal neurons (Figure 2C). These data show that it is feasible to use lentivirus to express TAP-tagged proteins in cultured neurons, subsequently purified by TAP protocol. There are limitations for TAP-tagged purification, such as: (A) transient interactions can be missed. (B) The transgene may be expressed in cells that don’t normally express the channel. (C) Associated proteins may be missed if they are conditional upon the specific type of neuron studied. The TAP-MS method for studying protein interactomes has been previously used *in vivo* (Fernandez et al., 2009; Volkel et al., 2010; Kanellopoulos et al., 2018). Knockin of a TAP tag to the target protein in animals has the advantage of recapitulating the natural expression of the protein, thereby limiting artifactual interactions. However, it requires gene-modified mouse generation requiring more time, effort, and costs.

During the purification of the protein complex, a weak lysis buffer will usually be used to preserve low-affinity binding proteins. Using the kit-provided weak lysis buffer and standard TAP protocol from Interplay mammalian TAP purification kit, we identified several proteins including some synaptic proteins (Table 1). To examine if TAP protocol works in a stronger lysis buffer to isolate high-affinity binding proteins, we used 1% Triton X-100 + 0.5% sodium deoxycholate as detergents and harvested more neurons than in a standard TAP protocol. The number of DPP6, DPP10, and KChIP1-4 peptides was reduced, while the number of Kv4.1, Kv4.3, and mGluR5 peptides stayed at a similar level as that in the standard protocol, if normalized by the number of Kv4.2 peptides (Table 1), supporting the notion that the stringency of lysis buffer determines the protein complex. DREAM/KChIP3 binds to GluN1 and negatively regulates GluN1 (Zhang et al., 2010), and they both were identified in our TAP-MS analysis using both weak and strong lysis buffer. Cav3 and Kv4 seem to form a complex in cerebellar granule cells (Heath et al., 2014). Cav3 is not identified by TAP-Kv4.2 using both weak and strong lysis buffer in hippocampal neurons, which may suggest that the protein complex is cell specific.

Kv4.2 functions as tetramers as demonstrated by the crystal structure (Kise et al., 2021). It is interesting to find that Kv4.1 and Kv4.3 are in the Kv4.2 complex (Table 1), which suggests that Kv4.2 can form heteromultimers with Kv4.1 and Kv4.3. Future studies should examine the differences in channel properties and/or expression between heteromultimers and homomultimers and if the role of auxiliary subunits (DPPs and KChIPs) in regulation of Kv4 heteromultimers is different compared to homomultimers.

It has also been reported that Kv4.2 functions together with mGluR5 and NMDA receptors (Hu et al., 2007; Kim et al., 2007). Kv4.2 activity remodels synaptic NMDA receptors by regulating the relative synaptic NR2B/NR2A subunit composition ratio at hippocampal synapses (Jung et al., 2008). Ablation of Kv4.2 in mice abolished the gradual reduction in GluN2B/GluN2A subunit ratio during post-natal development and resulted in a higher proportion of silent synapses in adulthood (Kim and Hoffman, 2012). Metabotropic glutamate receptor 5 regulates excitability and Kv4.2-containing K^+^ channels primarily in excitatory neurons of the spinal dorsal horn (Hu and Gereau, 2011). We identified that mGluR5 and GluN1/2B are in the Kv4.2 protein complex (Table 1) and validated that mGluR5 and GluN1/2B co-immunoprecipitated with Kv4.2 when expressed in HEK293T cells (Figures 3A–C) and co-localized with Kv4.2 in cultured hippocampal neurons (Figures 3D–F). These data support the notion that Kv4.2 functions in complex with glutamate receptors.

Taken together, our results reveal a method to identify protein complexes of neuronal proteins and PTMs of neuronal proteins. Future studies may use different neuronal types or age of neurons to identify variations in protein complexes of interest using this protocol. Furthermore, by choosing the strength of detergent in the lysis buffer, high- or low-affinity protein interaction can be determined and activity-induced PTMs measured.

## Data availability statement

The original contributions presented in this study are included in the article/Supplementary material, further inquiries can be directed to the corresponding author/s.

## Ethics statement

The animal study was reviewed and approved by the NICHD ACUC.

## Author contributions

J-HH and DH conceived the work. J-HH and YL performed the studies. J-HH, YL, and DH interpreted the results and wrote the work. DH provided supervision and acquired funding. All authors contributed to the article and approved the submitted version.

## Footnotes

1
https://www.uniprot.org/taxonomy/10116

## Supplementary material

Supplementary Table 1 Kv4.2 phosphorylation sites identified by TAP-MS in control condition. The table shows the score of each phospho-peptide that was detected in control condition. The Ascore values above 13 were labeled green, and above 19 (more confidently assigned for phosphorylation) were labeled red. If the Ascore values for Ascore Seq_A and Ascore Seq_B are both above 13 for the same site, then the location is considered confidently assigned. In the case of multiple phosphorylation events, the Ascore1 refers to the N-terminal most site and Ascore 2 refers to the next site moving toward the C-terminal. #: label the residue for phosphorylation analysis.

Supplementary Table 2 Kv4.2 phosphorylation sites identified by TAP-MS with AMPA treatment. Table shows the score of each phospho-peptide that was detected in AMPA (50 μM, 15 min) treatment condition. The Ascore values above 13 were labeled green, and above 19 (more confidently assigned for phosphorylation) were labeled red. If the Ascore values for Ascore Seq_A and Ascore Seq_B are both above 13 for the same site, then the location is considered confidently assigned. In the case of multiple phosphorylation events, the Ascore1 refers to the N-terminal most site and Ascore 2 refers to the next site moving toward the C-terminal. #: label the residue for phosphorylation analysis.
